# A96 IMPROVING AUTOMATED TELEHEALTH SERVICES TO MEET THE NEEDS OF PATIENTS AND HEALTHCARE PROFESSIONALS

**DOI:** 10.1093/jcag/gwac036.096

**Published:** 2023-03-07

**Authors:** L Calman T'ien, C Galorport, R A Enns

**Affiliations:** St. Paul's Hospital, Vancouver, Canada

## Abstract

**Background:**

There is a growing demand for automated telehealth programs in medicine to improve health-related behaviours such as adherence to treatment schedules. In addition, these services are a promising alternative to increase access to medical care for rural and marginalized patients.

Although promising, telehealth programs need to be evaluated based on patient responsiveness in order to tailor automated services to their target population and identify factors that minimize response rates.

**Purpose:**

To evaluate the effect of socioeconomic, health and structural factors on the response rate of an automated follow-up program implemented at St. Paul’s Hospital in Vancouver, BC.

**Method:**

A retrospective chart review was conducted of patients who did not respond when contacted by a PAtient-Guided Complication Tracking System (PACTS). PACTS sent Short Message Service (SMS) to outpatients having a flexible sigmoidoscopy, gastroscopy and/or colonoscopy one week post-procedure.

Patients who received PACTS SMS between 03/21-08/21 were included in this study. Individuals were considered non-responsive if they failed to reply after receiving an initial SMS and a second reminder text message (sent 24 hours after the first SMS).

Socioeconomic factors including: age, sex and personal annual income were assessed. Income was analyzed using postal code census data. To study health factors, patient comorbidity was evaluated using the Charlson Comorbidity Index (CCI) where CCI > 2 was considered high. Finally, access to a general practitioner (GP) was investigated to study structural factors influencing responsiveness to PACTS.

**Result(s):**

Of the 200 people studied, 109 of these individuals were male (54%) and 91 were female (46%). The mean age of non-respondents was 60.2 ± 16.4. Postal codes were reported for 144 patients (72%) and the mean annual income of these individuals was $48 928 ± $9710. The mean Charlson Comorbidity Index was 2.1 ± 1.7. 109 non-respondents (54%) had a family doctor listed in their chart and 91 non-respondents (46%) did not.

**Conclusion(s):**

Based on the age, sex, personal annual income and comorbidity results, socioeconomic and health factors do not impact response rate. The large number of non-respondents without GPs indicate that structural factors influence responsiveness. The high proportion of non-respondents lacking GPs may represent a subgroup of individuals that under-use healthcare services.

Further evaluation of non-respondents and comparative analysis with a large group of respondents are pending and will likely support these conclusions.

**Please acknowledge all funding agencies by checking the applicable boxes below:**

None

**Disclosure of Interest:**

None Declared

